# Supplementary material for: Methionine matters: a common mechanism of viral inhibition of host defense identified via AI-assisted molecular dynamics
Source: bioRxiv. 2025 Oct 8:2025.09.19.677134. Originally published 2025 Sep 19. Preprint. [Version 2] doi: 10.1101/2025.09.19.677134 (PMC12458179; doi:10.1101/2025.09.19.677134)
Supplement: 1 — Supplemental Figure 1. Root mean square fluctuation plots for the computational binding experiments on (A-B) VSV M, (C-D) KSHV ORF 10, (E) SARS-CoV-1 ORF 6 and (F) SARS-Cov-2 ORF 6. The bound vs unbound rmsf values are shown resp as red and black and appear stable at both 1ns (A,C,E and F) and 10ns (B, D) time intervals. Supplemental Figure 2. Site-wise average root mean square fluctuation plots comparing (A-B) bound vs unbound KSHV ORF 10 and VSV M structures and (C-D) Supplemental Figure 3. Site-wise denoised molecular dynamics comparisons (A-B) wild-type vs. D52G mutant Rae1-Nup98 bound VSV M protein. Here is shown the additional amplification in functional sites of the host Rae1-Nup98 (C-D) as it tries to hold onto the mutant viral ORF 6 that is lacking a key acidic flanking residue in the region required for binding. The method of denoising utilizes the maximum mean discrepancy (MMD) in learned features of local atom fluctuations that best distinguish the two dynamic states being compared (Babbit et al. 2024). As in Figure 2 the MMD is signed negative (blue) to indicate dampened motion and positive (red) to indicate amplified motion. Supplemental Figure 4. Site-wise denoised molecular dynamics comparisons (A-B) wild-type vs. M to R mutant Rae1-Nup98 bound SARS-CoV-1 ORF 6 protein. Here is shown the additional dampening in functional sites of the host Rae1-Nup98 (C-D) as it tries to hold onto the mutant viral ORF 6 that is lacking the key methionine region required for binding. The method of denoising utilizes the maximum mean discrepancy (MMD) in learned features of local atom fluctuations that best distinguish the two dynamic states being compared (Babbit et al. 2024). As in Figure 2 the MMD is signed negative (blue) to indicate dampened motion and positive (red) to indicate amplified motion. Supplemental Figure 5. Site-wise denoised molecular dynamics comparisons (A-B) wild-type vs. M to R mutant Rae1-Nup98 bound SARS-CoV-2 ORF 6 protein. Here is shown the ad [file NIHPP2025.09.19.677134V2-supplement-1.pdf]

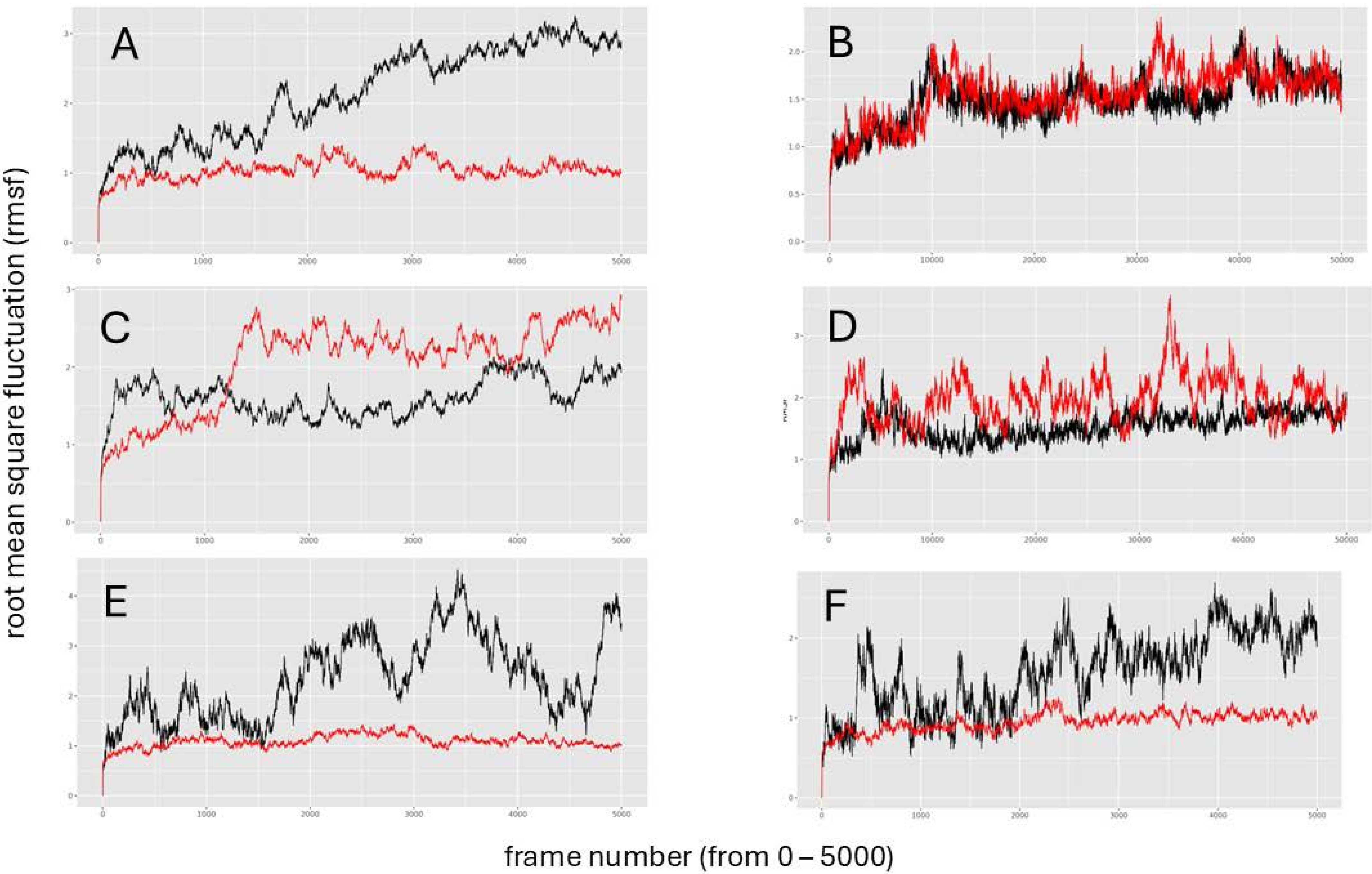
A
B
C
D
E
F
root mean square fluctuation (rmsf)
frame number (from 0 – 5000)

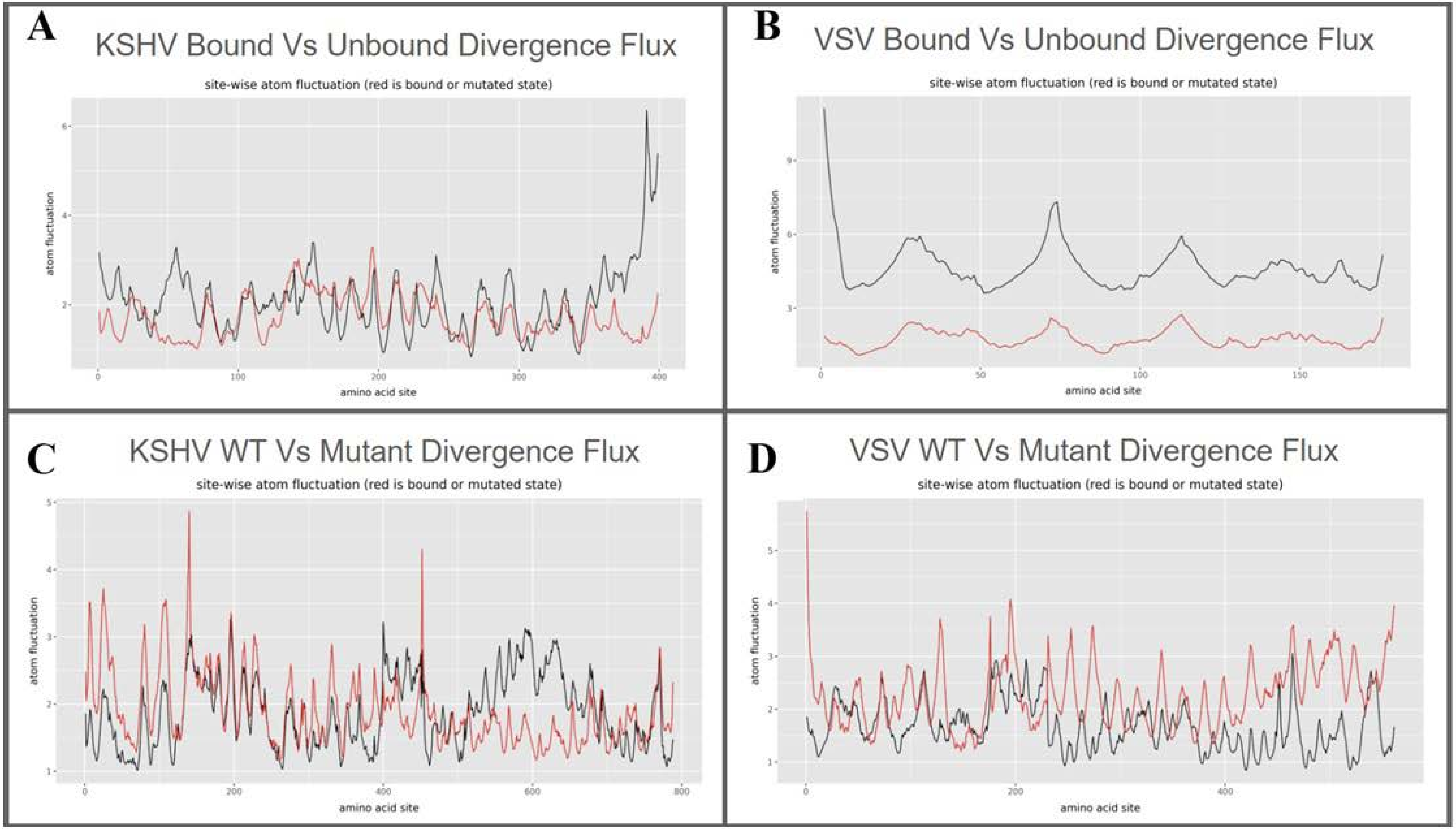
A
KSHV Bound Vs Unbound Divergence Flux
site-wise atom fluctuation (red is bound or mutated state)
atom fluctuation
amino acid site
B
VSV Bound Vs Unbound Divergence Flux
site-wise atom fluctuation (red is bound or mutated state)
atom fluctuation
amino acid site
C
KSHV WT Vs Mutant Divergence Flux
site-wise atom fluctuation (red is bound or mutated state)
atom fluctuation
amino acid site
D
VSV WT Vs Mutant Divergence Flux
site-wise atom fluctuation (red is bound or mutated state)
atom fluctuation
amino acid site

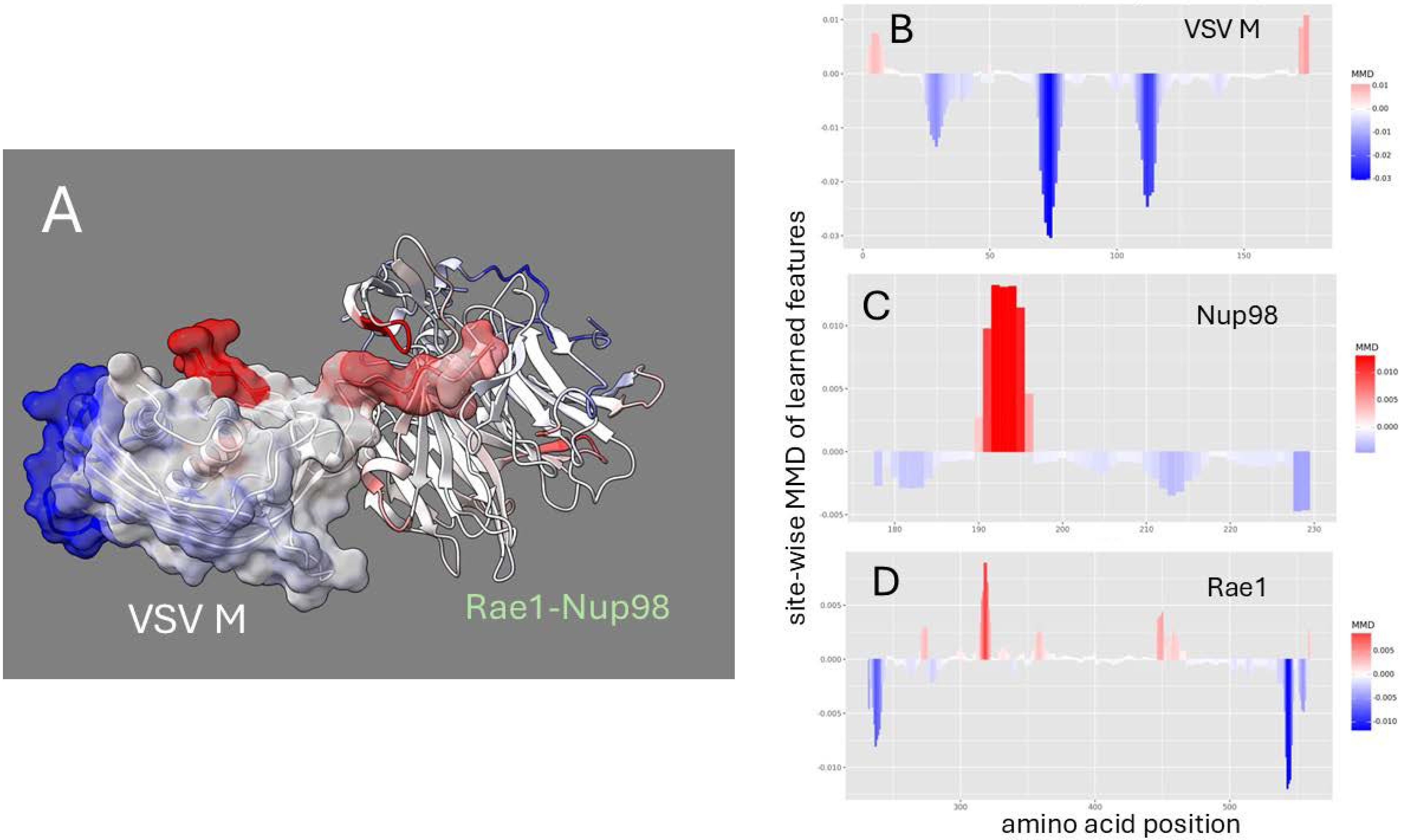
A
VSV M
Rae1-Nup98
B
VSV M
C
Nup98
D
Rae1
MMD
site-wise MMD of learned features
amino acid position

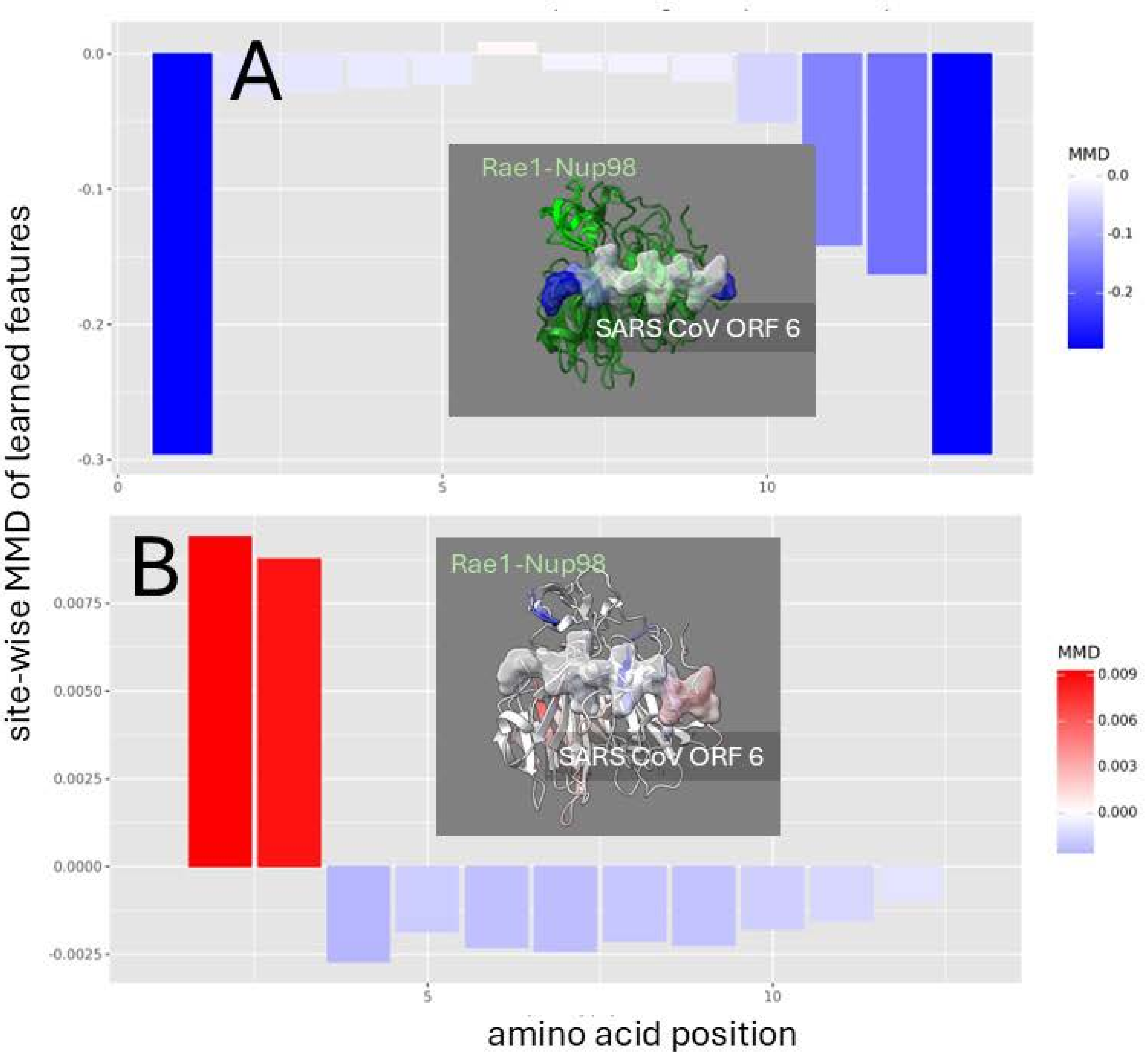
A
B
Rae1-Nup98
SARS CoV ORF 6
Rae1-Nup98
SARS CoV ORF 6
MMD
0.0
-0.1
-0.2
MMD
0.009
0.006
0.003
0.000
0.0
-0.1
-0.2
-0.3
0.0075
0.0050
0.0025
0.0000
-0.0025
0
5
10
5
10
site-wise MMD of learned features
amino acid position

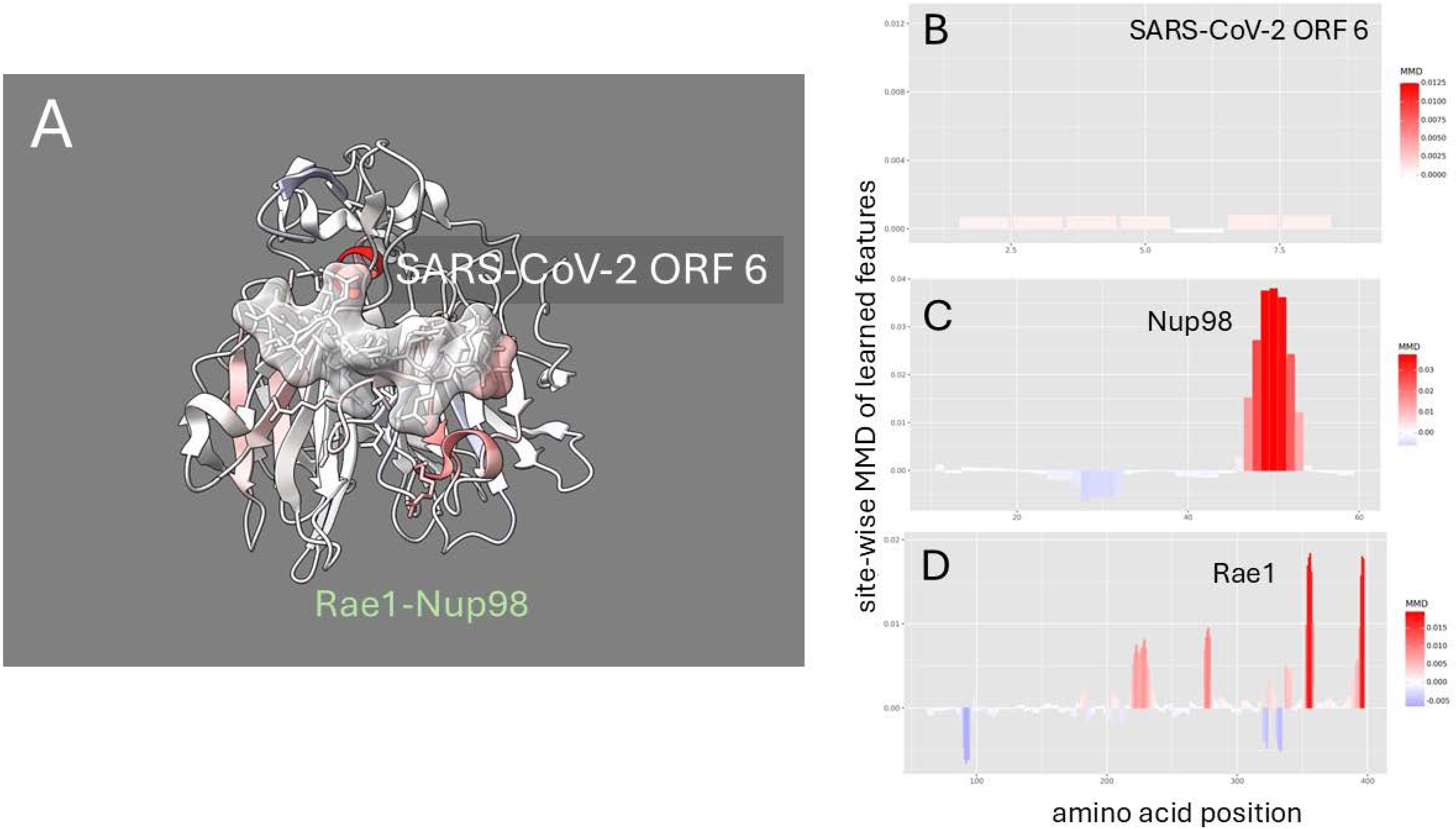
A
SARS-CoV-2 ORF 6
Rae1-Nup98
B
SARS-CoV-2 ORF 6
C
Nup98
D
Rae1
MMD
site-wise MMD of learned features
amino acid position

Protein	Interacting Binding Grove Residues										
SARS-CoV-2 ORF 6	54	E	E	Q	P	M	E	I	D		61
SARS-CoV-1 ORF 6	54	D	E	E	P	M	E	L	D	Y	62
VSV M	49			D	E	M	D	T	H	D	55
KSHV ORF10	409	G	E	S	P	M	E	W	Q	T	417
